# Vaccimel immunization is associated with enhanced response to treatment with anti-PD-1 monoclonal antibodies in cutaneous melanoma patients - a case reports study

**DOI:** 10.3389/fimmu.2024.1354710

**Published:** 2024-04-25

**Authors:** José Mordoh, Erika Schwab, Alicia Inés Bravo, Mariana Aris, María Marcela Barrio

**Affiliations:** Centro de Investigaciones Oncológicas, Fundación Cáncer (FUCA), Ciudad Autónoma de Buenos Aires, Argentina

**Keywords:** cutaneous melanoma, vaccine, case report, immune checkpoints inhibitors, immunotherapy

## Abstract

Cancer vaccines are gaining ground as immunotherapy options. We have previously demonstrated in cutaneous melanoma (CM) patients that adjuvant treatment with VACCIMEL, a mixture of four irradiated CM cell lines co-adjuvanted with BCG and GM-CSF, increases the cellular immune response to melanocyte differentiation antigens, cancer-testis antigens and neoantigens, with respect to basal levels. On the other hand, it is also known that treatment with anti-PD-1 monoclonal antibodies (MAbs), acting on pre-existing tumor-reactive lymphocytes, induces clinical responses in CM patients, albeit in a fraction of treated patients. A combination of both treatments would appear therefore desirable. In this paper, we describe CM patients who, having progressed even years after vaccination, were treated with anti-PD-1 MAbs. In 5/5 of such progressor patients, complete responses were obtained which lasted between 3 and 65+ months. Three of the patients remain disease-free and two recurred. One of the patients passed away after a recurrence of brain metastases. We suggest that clonally expanded reactive lymphocytes induced by VACCIMEL partially remain as memory cells, which may be recalled after tumor recurrence and may foster ulterior activity of anti-PD-1 MAbs.

## Introduction

Cutaneous melanoma (CM) is still a serious disease, due to its increasing incidence and its generally rapid growth and dissemination. Fortunately, during the last two decades, great advances have been made in its treatment. Such advances stem from the exploitation of two CM Achilles´ heels: its elevated tumor mutational burden (TMB), which gives rise to a large number of neoantigens (neoAgs) and the presence of mutated driver oncogenes, such as BRAF^V600^, in 50% of the patients. As a consequence, new treatments have become available, such as inhibitors of the MAPK pathway and of monoclonal antibodies (MAbs) inhibitors of the CTLA4/CD80 and PD-L1/PD-1 immune checkpoints inhibitors (ICI) [see Long et al. for a review (1)]. With respect to immunotherapy treatments, the mechanisms involved in their effectiveness and resistance are still incompletely understood. Concerning effectiveness, in a study of 655 metastatic CM patients treated in monotherapy with the anti-PD-1 MAb pembrolizumab (2), the overall survival (OS) after 5, years of follow-up was 34% for the total population. Besides, the complete response (CR) rate was only 16%, whereas the partial response (PR) rate was 25%, and stable disease (SD) accounted for 24% of the patients. Thus, 75% of the responding patients still had residual disease and were more prone to recurrence than those who attained CR. There is therefore an unsatisfied medical need to ameliorate this situation. Due to its high TMB, it is probable that during CM growth different cell clones are generated, which in turn express a great variety of neoAgs, only some of which are capable of triggering effective immune responses (3). Anti-PD-1 MAbs are usually administered without any previous treatment, and it is accepted that they act on already existing anti-tumor reactive T cell clones by reversing their exhaustion and increasing their cytotoxicity (3). To widen the immune system reactivity against a greater number of Tumor-Associated Antigens (TAA) and neoAgs, we have developed the CM allogeneic vaccine VACCIMEL, with BCG and GM-CSF as adjuvants. Two Phase I trials were performed on stages IIB-IV CM patients which demonstrated that the combination did not induce any high-grade toxicity (4, 5). Also, a randomized phase II study (CASVAC-0401) comparing VACCIMEL versus IFN-alpha2b in adjuvancy was performed in stages IIB, IIC and III CM patients (6). After 48 months of follow-up, the vaccinated patients had a significantly higher median distant-metastases free survival (DMFS) than those treated with IFN-alpha2b (96 months and 13 months, respectively). Also, VACCIMEL-treated stage III CM patients attained, at 48 months follow up, similar DMFS than patients treated with anti-PD1 MAbs (7). Using IFN-gamma ELISPOT assays we demonstrated that during the 2-year treatment, VACCIMEL induced an expansion of CD4^+^ and CD8^+^ T cells targeting TAA, such as melanocytic differentiation (MD)-Ags and cancer testis (CT)-Ags in all vaccinated patients. In some patients, we also observed an immune response to the patient`s private neoAgs, i.e. those expressed by the patient´s tumor, presumably by induction of antigen spreading in a strong Th1-polarized immunity microenvironment (8). Some patients who progressed after vaccination were treated with anti-PD-1 MAbs outside the clinical study, allowing us the opportunity to analyze the clinical responses and the possible toxicities of the combination. We report here 5/5 patients who progressed after vaccination and attained CR of different durations (3-65+ months) after anti-PD-1 treatment without any added toxicities.

## Cases description

**Figure 1:**
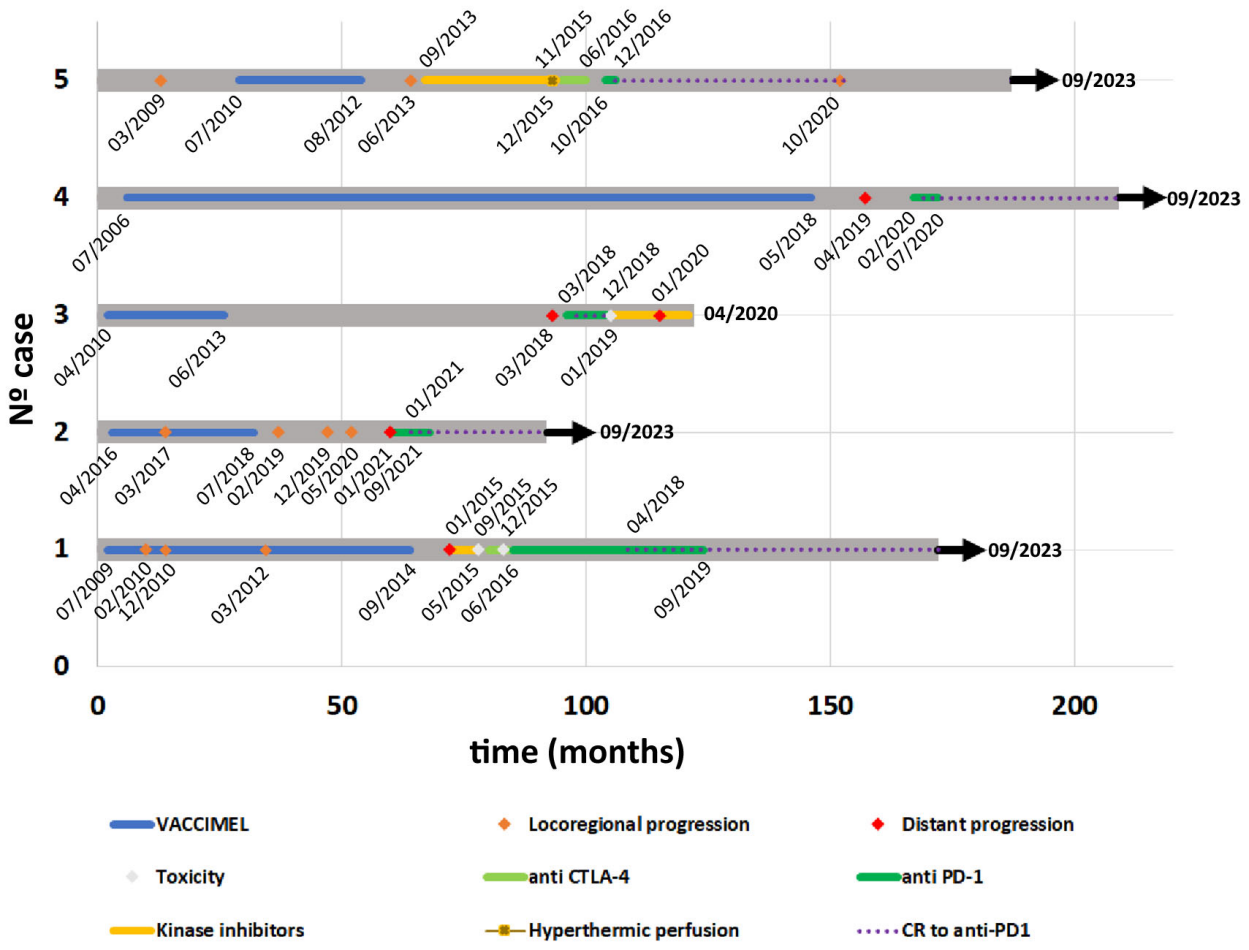
Timeline from all case reports. Course of the disease, expressed in months, and treatments received by each patient are illustrated with a swimmer plot. Relevant dates are indicated within the timeline. Color references for each kind of event are indicated in the legend. Case#1: after pembrolizumab treatment, this patient attained complete remission in 04/2018 (POST) and remained NED until present. Case#2: After pembrolizumab treatment, this patient attained complete remission (04/2021) and remained NED until present. Case#3: Although this patient achieved a rapid CR to nivolumab (05/2018), had to shift to dabrafenib/trametinib, because of a supraventricular arrhythmia (12/2018), with further progression. Case#4: After pembrolizumab treatment, this patient attained complete remission (05/2020) and remained NED until present (09/2023). Case#5: this patient remained NED 46 months after CR to nivolumab (12/2016), further described (7). CR, Complete Response; NED, no evidence of disease.

The patients #1 to #5 included in this Case Report were previously treated with VACCIMEL in different Phase I and Phase II clinical studies, approved by the Institutional Review Board of the Alexander Fleming Institute. All patients provided written informed consent for publication of his/her results. With the exception of patient #1, who had participated in a previous Phase I study, the rest of the patients were selected from a 30 patients cohort treated with VACCIMEL (7). From those patients, we have selected five progressor patients to metastatic disease, from whom we could obtain detailed clinical stories and the pertinent imaging studies. Those patients were treated at some point with anti-PD-1 MAbs, some of them among other treatments. The details of the disease course and treatments received are summarized in a swimmer plot (**Figure 1**, timeline).

The detailed clinical data of the patient´s tumors are described in **Supplementary Table 1**, and the sequence of patients’ evolution is described in **Supplementary Table 2**.

**Figure 2:**
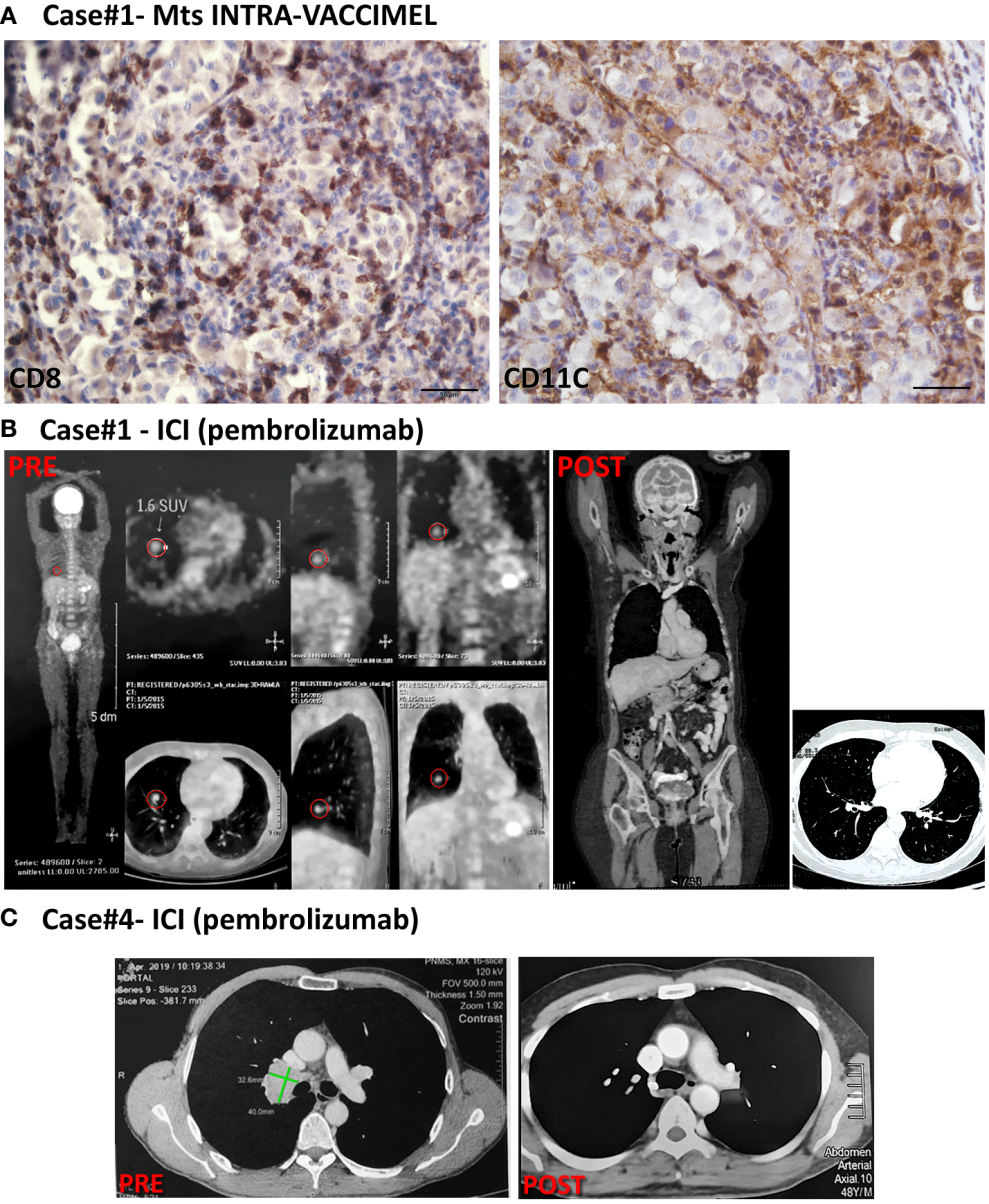
Patient #1 and #4 cases. **(A)** IHC of an inguinal LN metastasis excised from case#1 intra-VACCIMEL treatment; CD8^+^ and CD11c^+^ populations are shown. IHC was performed as previously described (9). Original magnification 400X; scale bars 50µm. **(B)** PET/CAT scan from case#1 (PRE, 01/2015) showed hypercaptating right lung nodules and a parasplenic nodule. After pembrolizumab treatment, this patient attained complete remission in 04/2018 (POST) and remained NED until present (09/2023). **(C)** CT from case#4, dated 04/2019 (PRE) showing lung right lung hilar metastases– (indicated by green lines), not detectable after treatment with Pembrolizumab (POST; dated 07/2020).

Case #1 is a 65-year-old white Caucasian female to whom in 1993 an inferior left dorsal CM was excised. In 04/2009 (**Figure 1**, timeline) she started vomiting, and in 05/2009 a segmentary resection of the small intestine was performed. An intramural CM metastasis was found and 3/9 analyzed lymph nodes (LNs) were also metastatic. Histological analysis of the small intestine metastasis revealed an amelanotic CM without lymphoid infiltration (*data not shown*). Therefore, the patient was at stage IV of her disease. A PET scan performed in 06/2009 revealed residual disease at cervical LNs, mesenteric root LNs, and subcutaneous metastasis in the left gluteus. In 07/2009 the patient entered a phase I clinical study of autologous dendritic cells (DC) loaded with VACCIMEL, but she could only receive 3 doses of 10 x 10^6^ DC loaded with apoptotic–necrotic tumor cells, 2 weeks apart, since the yield of PBMC after leukapheresis was low. The patient was therefore shifted to receive VACCIMEL plus BCG plus GM-CSF every two months. In 02/2010 an intra-treatment inguinal adenopathy appeared that was resected. Histologic analysis revealed a metastatic lymph node with brisk tumor infiltration with CD8^+^ lymphocytes and a high number of CD11c^+^ DC (**Figure 2A**). In 12/2010 and 03/2012 a para-psoas LN and a gluteus dermic metastases were excised. Between 07/2009 and 09/2014 the patient received 23 vaccinations. In 01/2015 a PET/CT scan revealed several hypercaptating nodules at both lungs and two hypermetabolic para-splenic and para-pancreatic nodules (**Figure 2B** PRE). Since the patient´s tumor had the BRAF^V600E^ mutation, she was treated with vemurafenib between 01/2015 and 05/2015, at which time it was permanently suspended because of grade III dermic toxicity and tumor progression. Starting in 09/2015 she received five courses of Ipilimumab (3 mg/kg every three weeks), and in 06/2016 she started treatment with pembrolizumab (2 mg/kg every three weeks), which she continued until 09/2019, without significant toxicity (see timeline in **Figure 1**). The patient attained CR in 04/2018 and remains disease-free until 09/2023 (65 months+) (**Figure 2B** POST). The period between the end of vaccination and the start of pembrolizumab was 21 months.

**Figure 3:**
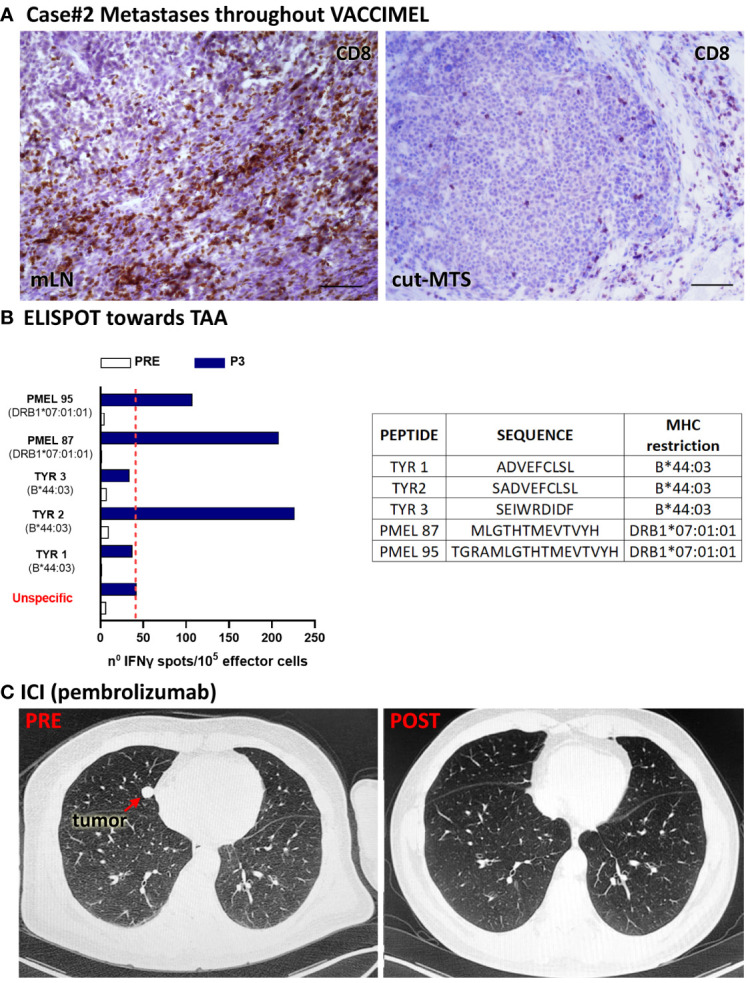
Patient #2 case. **(A)** Histopathological analysis of a tumor draining LN and a cutaneous metastases excised throughout VACCIMEL immunization and 4 years before anti-PD-1 treatment; CD8+ populations as determined by IHC are shown. Original magnification 400X; scale bars 50µm. **(B)** IFN-γ ELISPOT to analyze T-cell response induced by VACCIMEL (8). PRE: blood extracted at the selection process; POST-3: blood extracted 24 months after protocol start; unspecific: corresponds to PBMC only stimulated with culture medium. Bars indicate quantification of the spots normalized to 10^5^ stimulated PBMC (effector cells). Peptides tested for this case are shown in the table. **(C)** PRE: CAT scan showing a lung metastasis (red arrow), non-detectable after ICI treatment with pembrolizumab (POST).

Case #2 is a 59-year-old, white Caucasian male. Since 2006, he has been allergic to undefined allergens, with sneezing and dyspnea. In 01/2016 an epithelioid CM was excised from his back. The lesion was in a vertical growth phase, had a Breslow index of 2.3 mm, it was not ulcerated and had an intermediate mitotic index. Lymphoid infiltration was not detected. A sentinel lymph node biopsy (SLNB) in the right axilla was performed and a micro-metastatic LN was found which contained gp100^+^ and Melan-A^+^ cells (*not shown*). The patient was at stage IIIA and radical LN dissection was not performed at that time. In 04/2016 he signed the informed consent to enter the CASVAC-0401 study (**Figure 1**, timeline). In 08/2016 two cutaneous metastatic lesions close to the primary tumor scar appeared and were excised. The patient continued in the study as allowed per protocol. From 11/2016 onward the BCG doses in VACCIMEL were reduced to 2x10^5^ CFU per vaccination due to intense local reaction. In 03/2017, a dermic nodule in the upper right scapular area and an enlarged right axillary LN were detected and biopsied (**Figure 3A**). In both cases, metastases were found and excised in 05/2017, after a PET/CT scan revealed no distant metastases. Histopathological analysis of LN revealed brisk CD8^+^ infiltration and the excised metastasis revealed a portion heavily infiltrated by CD8^+^PD1^+^ cells and another part of the same nodule with almost no lymphoid infiltration (9).

The patient continued in the study and ended his participation in 07/2018 without further events. Noteworthy, peripheral blood mononuclear cells (PBMC) increased IFN-γ release to TAA following immunization (**Figure 3B**). In 1/2021, a CAT-Scan revealed a right paracardiac nodule (17 mm diameter) and a micronodule (3 mm diameter) in the upper left lung lobule. The patient, whose tumor was WT for BRAF oncogene, started treatment in 01/2021 with pembrolizumab (200 mg every 3weeks). Therefore, 29 months elapsed since the end of VACCIMEL treatment and start of pembrolizumab treatment. In 05/2021 the right paracardiac nodule reached 2.4 cm diameter and the pulmonary nodule increased to 1.2 cm, as detected by PET-CAT scan (**Figure 3C** PRE). After 12 infusions, a new CAT-Scan was performed in 09/2021 and a CR of the lung (**Figure 3C** POST) and paracardiac nodules was observed. The patient has remained disease-free until 09/2023, when data were locked for this study. Thus, CR has so far lasted 24 months.

**Figure 4:**
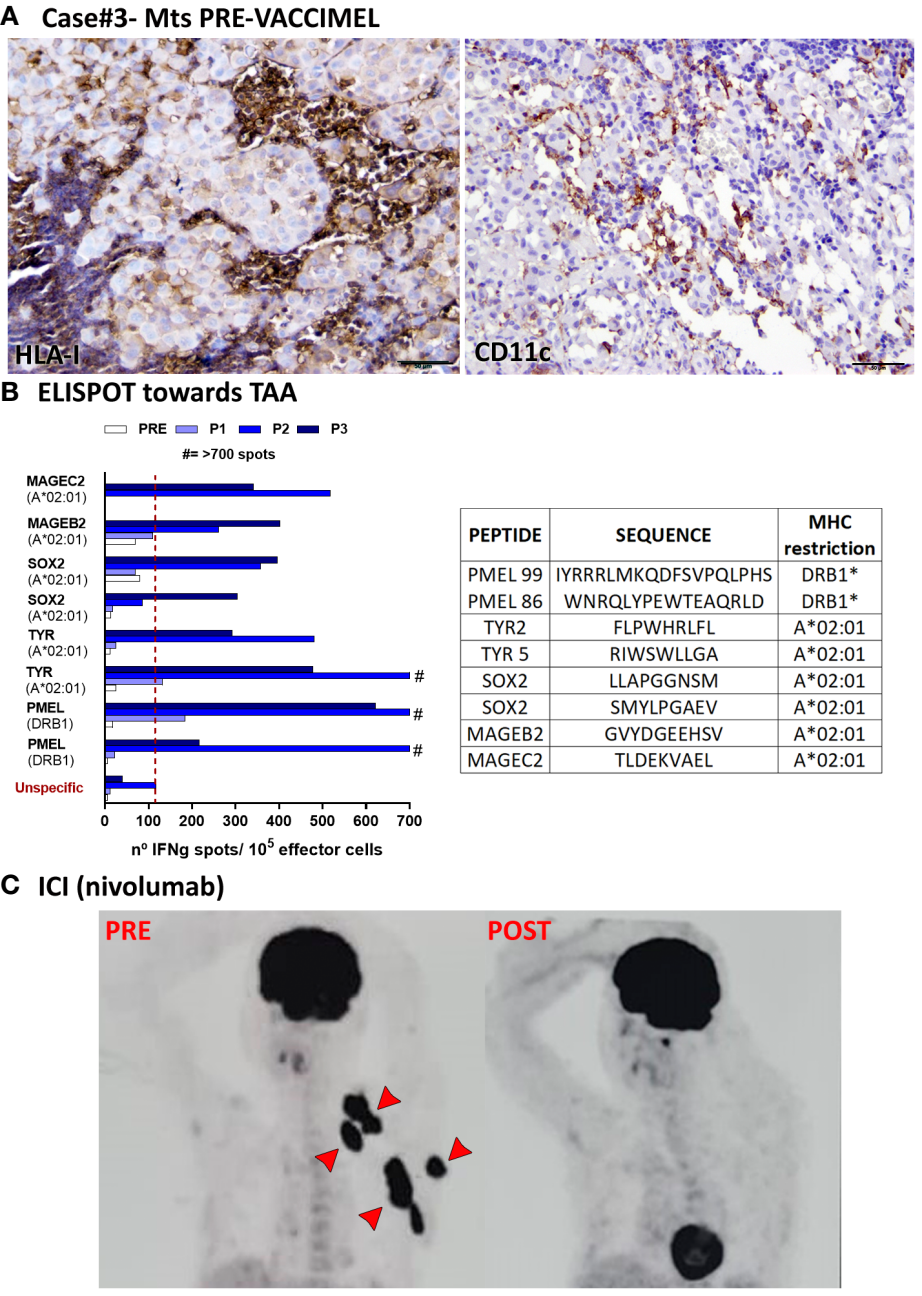
Patient #3 case. **(A)** Histopathological analysis of a LN metastasis excised prior to VACCIMEL immunization (03/2010). HLA-I^+^ and CD11c^+^ populations determined by IHC are shown. Original magnification 200X; scale bars 100µm. **(B)** T-cell response induced by VACCIMEL protocol to HLA-restricted peptides from shared melanoma associated antigens detected by IFN-γ ELISPOT was performed as described (8). Pre: blood extracted at the selection process; Post 1, 2 and 3 (P1, P2, P3): blood extracted 6, 12 and 24 months after protocol start; unspecific: corresponds to PBMC only stimulated with culture medium. Bars indicate quantification of the spots normalized to 10^5^ stimulated PBMC (effector cells). Peptides tested for this case are shown in the table. **(C)** A PET/CAT scan performed in 03/2018 showed multiple metastatic lesions (PRE), non-detectable after ICI treatment with nivolumab (09/2018) (POST).

Case #3 was a 49-year-old white Caucasian male, to whom in 01/2010 a dorsal melanoma was excised. The tumor was an ulcerated nodular polypoid melanoma, in vertical growth phase; Breslow index 4.1 mm; 2 mitosis/sq mm. Lymphoid infiltration was non-brisk, and satellitosis was present. In 03/2010, SLNB was performed, and 1/3 LN was found to be metastatic. This was followed by radical lymphadenectomy, which was negative for metastases (0/11 LN). Therefore, the patient was at stage IIIC of the disease. Histological analysis of the LN metastasis revealed scarce infiltration of tumor nests by CD8^+^ cells (*not shown*); HLA-I negative tumor cells were abundant (**Figure 4A** left) and CD11C^+^ cells were scarce (**Figure 4A** right). This patient had therefore bad prognostic features (10). On 26/04/2010 the patient entered the CASVAC 0401 study (6), and he was randomized to the vaccine arm (**Figure 1**, timeline). After receiving 13 vaccinations with VACCIMEL plus BCG plus GM-CSF, the patient ended NED (no-evidence of disease) the 2-year protocol in 05/2012 and received two additional vaccinations in 11/2012 and 06/2013. Reactivity to TAA increased throughout immunization, as determined by IFN-γ release from PBMC response in ELISPOT assays, thus revealing immune capabilities (**Figure 4B**). He remained NED until 03/2018 (52 months later), when clinical progression with left supraclavicular adenopathies, muscular and subcutaneous metastases, a nodule in the basal right lung and several small brain metastases were detected (**Figure 4C**, PRE). Three small brain lesions were treated in 03/2018 with gamma-knife surgery, and he started nivolumab treatment at the dose of 3mg/kg every three weeks, which he continued until 12/2018. A new PET/CAT Scan performed in 09/2018 revealed that the patient achieved a CR of the previous lesions (**Figure 4C** POST); the etiology of an increased isotope uptake observed at the root of the ascending aorta and the right atrium was not ascertained. In 12/2018 the patient´s brain metastases recurred and since the patient´s tumor had the BRAF^V600E^ mutation, the treatment was shifted to dabrafenib/trametinib in 01/2019. The duration of the CR was therefore 3 months and the patient passed away in April 2020. The period between the end of vaccination and start of nivolumab treatment was 57 months.

Case #4 is a 56 years-old white Caucasian male to whom in 2006 a melanoma in the retro-auricular right pavilion was excised (**Figure 1**, timeline). The lesion had a 6mm Breslow index and it was ulcerated. SLNB was performed, and a micrometastasis was found. Radical cervical LN resection was performed and no further metastatic LN were found (0/15 LN). A CAT-Scan revealed no metastatic disease. Therefore, this patient was at stage IIIC of his disease. In 07/2006 VACCIMEL treatment was started with the addition of GM-CSF 120 mg id for four days and BCG (0.5 x 10^6^ CFU per vaccination). From 08/2007 BCG doses were reduced and then suspended due to ulceration at the vaccination site. Between 08/2007 and 07/2008, he continued vaccination every three months without BCG, and then once a year until 05/2018, having no evidence of disease, twelve years after SLNB. However, a control CAT scan performed in 04/2019 revealed a 41 x 22 mm right lung hilar adenopathy determining an interval of 11 months between the end of vaccination and the appearance of metastasis. A PET scan confirmed the lesion but no other metastases were found (*not shown*). In 07/2019 a VATS surgery was performed, a biopsy was obtained and revealed melanoma metastasis, which had the BRAF^V600E^ mutation. Due to the COVID-19 pandemic, this patient could only start on 02/2020 treatment with pembrolizumab (200 mg every 3 weeks), of which he could only receive three infusions because of travel restrictions (**Figure 2C**, PRE). Therefore, the interval between the last vaccination and the start of pembrolizumab was 21 months. In 07/2020 a new CAT-Scan was performed and revealed CR of the hilar adenopathy (**Figure 2C**, POST). He received no further treatment and remains disease-free for 38 months until the time this report is written.

Case #5 is a 47-year-old, white Caucasian female, to whom in 02/2008 a CM was excised from her right leg. Two years after surgery, satellite lesions appeared and were excised; inguinal LN were spared. On 07/10 the patient entered the CASVAC 0401 study and she was randomized to the VACCIMEL arm (**Figure 1**, timeline). This patient`s case was described in detail in a previous paper from our group (7). After responding to VACCIMEL during 30 months, the patient progressed locally and was treated with vemurafenib, and ipilimumab, which response was an increase in the number of reddish lesions in her right leg and the treatment was interrupted. The patient also received hyperthermic perfusion with a CR, followed one year later by reappearance of the lesions. After relapse, she received nivolumab, administered at 300 mg every three weeks, which induced a CR that lasted 48 months before recurrence, which incidentally took place in the posterior part of the leg whereas previous lesions were mostly located in the inner part. Thirteen years after entering the CASVAC 0401 study, the patient is alive and well, with small lesions still confined to her right leg.

## Discussion

As previously described in the Introduction, the main purpose of this work was to inquire if the combination of VACCIMEL with anti-PD-1 MAbs increased the clinical responses or provoked special toxicities.

We demonstrated that 5/5 vaccinated patients who progressed even years after ending vaccination, responded with CR to anti-PD-1 treatment. We have not enough data to attribute such rate of CR exclusively to previous treatment with VACCIMEL since only patients #2, #3 and #4 received pembrolizumab/nivolumab as first-line treatment after detection of their metastasis. Patients #1 and #5 received ipilimumab prior to anti-PD-1; in the case of patient #5 this was accompanied by local disease progression. Bulk tumors were present in patients #1 and #3; the rest of the patients had small lesions. The case of patient #2 is of special interest since it was shown that different metastases simultaneously excised had diverging lymphoid infiltration (**Figure 3**) and (9). A reasonable hypothesis to put forward is that this patient has a few dominant tumor clones with the ability of metastasize: some clones would express the adequate Ags repertoire that makes them sensitive to cytotoxic lymphocytes; other clones would be “silent” with respect to Ags expression and therefore resistant to CD8^+^ cells.

With respect to patient #3, who has the shortest CR, it should be mentioned that his tumor cells had a quite low HLA-I expression; therefore it is not surprising that even when the patient was able to build immunity, the HLA-I^neg^ target cells would be resistant (**Figure 4**).

It is generally accepted that anti-PD-1 MAbs act by relieving immune suppression exerted by tumor cells or by the immune microenvironment through the PD-1/PDL-1 axis (11). As it refers to the intratumoral mechanism of action of anti-PD-1 MAbs *in vivo*, the evidence is contradictory. Ahmazadeh et al. (12) analyzed in 28 pretreated metastatic CM patients, lymphocytes purified from dissociated tumors (tumor infiltrating lymphocytes, TIL) and from normal adjacent tissues. They found that the majority of TIL, opposite to lymphocytes from normal tissues, expressed PD-1^+^CTLA-4^+^HLA-DR^+^Ki67^+^CD127^-^, a phenotype that the authors assume characterizes exhausted lymphocytes. Divergently, Ribas et al. (13) have studied 102 biopsies from 53 metastatic CM patients, obtained before treatment (basal) and during treatment with pembrolizumab. They found that PD-1 blockade increased the frequency of T cells, B cells, and myeloid-derived suppressor cells in responding patients. They also found that the predominant TIL from responding patients had the phenotype CD8^+^CD4^-^CD45RO^+^, corresponding to a memory phenotype. Surprisingly, they could not find CD8^+^ cells with an effector phenotype, and PD-1 expression was quite low in basal samples from responding and non-responding patients. Yost et al. analyzed TIL populations in patients with basal cell carcinoma and squamous cell carcinoma pre- and post-treatment with anti-PD-1 MAb, and concluded that the predominant TIL population in tumors after treatment was not that pre-existing before treatment, but rather a new clonal population of lymphocytes newly arrived into the tumors (14). Using an experimental system, Pauken et al. analyzed in mice infected with the influenza virus, which was the fate of CD8^+^ lymphocytes in PD-1 WT or KO mice (15). They found that the presence of WT PD-1 in CD8^+^ cells led to a restriction of cell proliferation in the early effector phase, this restriction leading to a high amount of memory cells. Instead, KO PD-1 CD8^+^ lymphocytes originated a stronger proliferation in the effector phase but a diminution of memory cells, and therefore a lesser recall activity. These findings suggest that a complicated interrelationship between the length of anti-PD-1 treatment and clinical results in patients may exist. A puzzling feature of anti-PD1 treatment is why, in cases in which SD or PR are the clinical outcomes, the tumor control is maintained even when the antibody has disappeared from the blood (16). This outcome may have a reasonable explanation if a pathological CR is obtained, since the disappearance of every tumor cell could explain the lack of recurrences. However, as before mentioned (2), in the pembrolizumab KeyNote 001 study, the ORR (overall response rate) was composed of 16% of patients attaining CR; 24% attaining PR and 25% attaining SD. Response was ongoing in 89% of patients who achieved CR and in 63% of patients who achieved PR. This suggests that even in patients who attain PR or SD, in whom tumor cells are presumably still present, immune activity is present long after pembrolizumab was cleared from blood, since the plasmatic concentration of pembrolizumab has a t1/2 of about three weeks. These results also highlight the importance of achieving CR in treated patients. As to the mechanism by which prior vaccination could enhance the effect of anti-PD-1 MAbs, we have previously demonstrated that VACCIMEL treatment induces T cell clones directed against TAA, such as MD-Ags and neoAgs, and which were usually non-detected before vaccination (8, 9, 17). We hypothesize that part of these responding T cell clones could become memory cells, probably located in secondary lymphoid organs, from which they would be recalled after metastasis reappearance and reinvigorated with anti-PD1 treatment even years after vaccination.

Although the number of cases were reported is small, it is interesting that we observed CR of different durations in 5/5 of the patients analyzed, lasting 65+; 24+; 3; 38+ and, 48 months. However, it should be signaled that two patients (#3 and #5) recurred and that patient #3 died from brain metastases. We suggest that VACCIMEL treatment previous to anti-PD-1 administration is a sequential combination that should be further explored.

## Data availability statement

The original contributions presented in the study are included in the article/**Supplementary Material**. Further inquiries can be directed to the corresponding author.

## Ethics statement

The studies involving humans were approved by Instituto Alexander Fleming Ethics Committee. The studies were conducted in accordance with the local legislation and institutional requirements. Written informed consent for participation in this study was provided by the participants’ legal guardians/next of kin. Written informed consent was obtained from the individual(s) for the publication of any potentially identifiable images or data included in this article.

## Author contributions

JM: Conceptualization, Funding acquisition, Investigation, Writing – original draft. ES: Investigation, Methodology, Writing – review & editing. AIB: Investigation, Methodology, Writing – review & editing. MA: Data curation, Investigation, Validation, Writing – original draft. MMB: Data curation, Investigation, Validation, Writing – review & editing.

## Supplementary material

Supplementary Table 1 Characteristics of the primary tumors from patients. NA: Not Available.

Supplementary Table 2 Main events of the disease and treatments received by all patients. ICI: Immune Checkpoint Inhibitors; PR: Partial Response: CR: Complete Response: PD: Progressive Disease. *Time of diagnosis of the primary tumor, except for case#1, who received VACCIMEL treatment following resection of an intestinal metastasis. **Duration of CR to anti-PD1 treatment.
