# Supplementary material for: Vaccimel immunization is associated with enhanced response to treatment with anti-PD-1 monoclonal antibodies in cutaneous melanoma patients - a case reports study
Source: Front Immunol. 2024 Apr 25;15:1354710. doi: 10.3389/fimmu.2024.1354710 (PMC11079628; doi:10.3389/fimmu.2024.1354710)
Supplement: Supplementary Table 1 — Characteristics of the primary tumors from patients. NA: Not Available. [file Table_1.docx]

| **Patient** | **Primary Tumor site** | **Melanoma**  **Type** | **Breslow**  **(mm)** | **Ulceration** | **Year of diagnosis** | **BRAF status** |
| --- | --- | --- | --- | --- | --- | --- |
| Case #1 | Inferior left back | NA | NA | NA | 1993 | V600E |
| Case #2 | Back | Epithelioid | 2.3 | no | 2016 | WT |
| Case #3 | Back | Nodular polypoid | 4.1 | yes | 2010 | V600E |
| Case #4 | Neck right side | Nodular | 6 | yes | 2006 | V600E |
| Case #5 | Right leg | Epithelioid extensive superficial | 0.89 | no | 2008 | V600E |

**Supplementary table 1. Characteristics of the primary tumors from patients.** NA: Not Available.
