# Supplementary material for: Vaccimel immunization is associated with enhanced response to treatment with anti-PD-1 monoclonal antibodies in cutaneous melanoma patients - a case reports study
Source: Front Immunol. 2024 Apr 25;15:1354710. doi: 10.3389/fimmu.2024.1354710 (PMC11079628; doi:10.3389/fimmu.2024.1354710)
Supplement: Supplementary Table 2 — Main events of the disease and treatments received by all patients. ICI: Immune Checkpoint Inhibitors; PR: Partial Response: CR: Complete Response: PD: Progressive Disease. *Time of diagnosis of the primary tumor, except for case#1, who received VACCIMEL treatment following resection of an intestinal metastasis. **Duration of CR to anti-PD1 treatment. [file Table_2.docx]

|  | **Case 1** | **Case 2** | **Case 3** | **Case 4** | **Case 5** |
| --- | --- | --- | --- | --- | --- |
| Diagnosis* | 05/2009 | 01/2016 | 03/2010 | 03/2006 | 02/2008 |
| VACCIMEL | 07/2009 09/2014 | 04/2016 07/2018 | 04/2010 05/2012 | 07/2006 05/2018 | 07/2010-08/2012 |
| Locoregional progression | 03/2010 | 03/2017, 02/2019, 12/2019, 05/2020 | - | - | 03/2009, 06/2013, 10/2020 |
| Distant progression | 05/2015 | 01/2021 | 03/2018, 01/2020 | 10/2019 | - |
| BRAF/MEK inhibitors | 05/2015 08/2015 (toxicity) | - | 01/2019 04/2020 (PD) | - | 09/2013 11/2015 (resistance) |
| Hyperthermic perfusion | - | - | - | - | 12/2015 (CR) |
| ICI (anti-CTLA-4) | 09/2015 12/2015 (toxicity) | - | - |  | 5 (PD) |
| ICI (anti-PD1) | 06/2016 09/2019 | 01/2021 09/2021 | 03/2018 12/2018 | 02/2020 07/2020 | 10/2016 12/2016 |
| CR to ICI (anti-PD1)** | 04/2018 09/2023  (65+ m) | 09/2021  09/2023  (24+ m) | 9/2018 12/2018  (3 m) | 07/2020 09/2023  (38+ m) | 12/2016 10/2020  (48 m) |
| Decease | - | - | 04/2020 | - | - |

**Supplementary table 2.** Main events of the disease and treatments received by all patients. ICI: Immune Checkpoint Inhibitors; PR: Partial Response: CR: Complete Response: PD: Progressive Disease. *Time of diagnosis of the primary tumor, except for case#1, who received VACCIMEL treatment following resection of an intestine metastasis. **Duration of CR to anti-PD1 treatment. m, months.
